# Vestibular dysfunction in Parkinson’s disease: a neglected topic

**DOI:** 10.3389/fneur.2024.1398764

**Published:** 2024-05-23

**Authors:** Meilin Gui, Lingling Lv, Lixia Qin, Chunyu Wang

**Affiliations:** ^1^Department of Neurology, The Second Xiangya Hospital, Central South University, Changsha, China; ^2^China National Clinical Research Center on Mental Disorders, Changsha, China; ^3^Department of Medical Genetics, The Second Xiangya Hospital, Central South University, Changsha, China; ^4^Key Laboratory of Hunan Province in Neurodegenerative Disorders, Central South University, Changsha, China

**Keywords:** dizziness, neuropathology, Parkinson’s disease, quality of life, vestibular system

## Abstract

Dizziness and postural instability are frequently observed symptoms in patient with Parkinson’s disease (PD), potentially linked to vestibular dysfunction. Despite their significant impact on quality of life, these symptoms are often overlooked and undertreated in clinical practice. This review aims to summarize symptoms associated with vestibular dysfunction in patients with PD and discusses vestibular-targeted therapies for managing non-specific dizziness and related symptoms. We conducted searches in PubMed and Web of Science using keywords related to vestibular dysfunction, Parkinson’s disease, dizziness, and postural instability, alongside the reference lists of relevant articles. The available evidence suggests the prevalence of vestibular dysfunction-related symptoms in patients with PD and supports the idea that vestibular-targeted therapies may be effective in improving PD symptoms.

## 1 Introduction

Parkinson’s disease (PD) is a neurodegenerative disease with a slow rate of progression primarily characterized by movement disorders, including resting tremors, rigidity, and bradykinesia. These symptoms often stand out prominently and have consistently been the primary focus of attention and treatment for most healthcare professionals. In contrast, dizziness and balance disorders are often overlooked as common symptoms in the elderly. Nevertheless, the prevalence of dizziness in patients with PD is twice higher than in normal elderly individuals (1), and the prevalence of balance disorders is indisputably higher, which greatly affects their quality of life (QoL) and urgently requires adequate attention and treatment.

The vestibular system, which integrates visual, proprioceptive, and vestibular signals, is the largest sensory system in the human body and is crucial for maintaining postural equilibrium and spatial orientation (2). Vestibular dysfunction is prevalent in PD (3–6) increasing the risk of falls (2, 7, 8). It is associated with dizziness, imbalance, gaze instability, and spatial disorientation, significantly affecting the QoL of patients (9, 10). The symptoms associated with vestibular dysfunction in patients with neurodegenerative diseases, including impaired balance, dizziness, and spatial disorientation, have recently been discussed (10). In addition, the anatomical and functional correlations between PD and vestibular system dysfunction have also been assessed (4). However, few reviews have focused on vestibular dysfunction-associated symptoms in patients with PD. Moreover, by integrating vestibular assessment and targeted therapeutic approaches into clinical practice, healthcare professionals can provide more nuanced and comprehensive care for patients with PD. Therefore, in this review, we summarize the clinical characteristics of vestibular dysfunction-related symptoms in patients with PD and explore vestibular-targeted therapies for nonspecific dizziness of PD with the aim of bridging the gap between identifying vestibular dysfunction and its effective management in PD.

## 2 Symptoms of vestibular dysfunction in patients with PD

**Figure 1:**
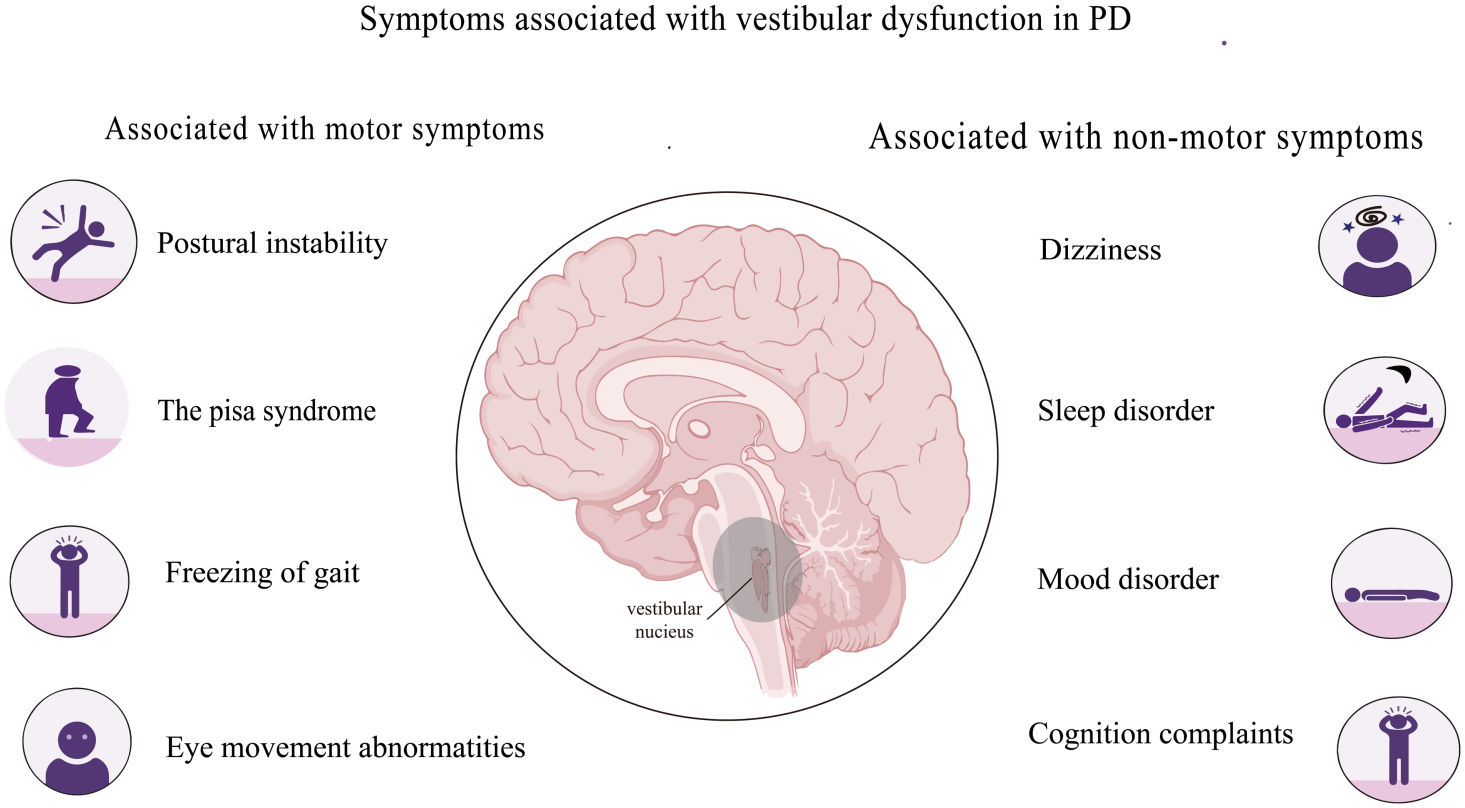
Symptoms associated with vestibular dysfunction in PD.

Vestibular dysfunction in PD might be associated with motor symptoms, including postural instability, Pisa syndrome, freezing of gait, abnormal eye movement, and non-motor symptoms such as dizziness, sleep disturbances, mood abnormalities, and cognitive impairment (Figure 1).

### 2.1 Associations between vestibular dysfunction and motor symptoms of PD

Resting tremors, limb rigidity, bradykinesia, postural abnormalities, gait dysfunction, and axial symptoms are established markers of disability in patients with PD. They usually manifest as a stooped or externally flexed posture, with reduced or increased variability in stride length and gait speed. As the main sensory system in maintaining balance and gait, the vestibular system might be involved in the mechanisms that drive such anomalies. Accordingly, vestibular laboratory tests have substantiated various such abnormalities in patients with PD (Table 1).

**Table 1:** Summary of studies associated with abnormal vestibular laboratory findings in Parkinson’s disease (PD).

Study	Groups	Outcome measure	Proportion of patients with each characteristic
Venhovens et al. (2)	Patients with PD (*n* = 30) Patients with AP (*n* = 14) HCs (*n* = 25)	oVEMPs, cVEMPs, BAEPs, SVV, VNG	Evidence of vestibular dysfunction in laboratory examinations in patients with PD (90%) Falling at least once per year (37%) Orthostasis (27%) Postural instability (82%) FoG (45%)
Bohnen et al. (3)	Patients with PD (*n* = 106) HCs (*n* = 29)	The six conditions of the SOT	Disequilibrium in patients with PD (68%)
Shalash et al. (7)	Patients with PD (*n* = 15) HCs (*n* = 15)	oVEMPs, cVEMPs, BAEPs	Absence of ACS oVEMPs in patients with PD (47%) Absence of ACS cVEMPs in patients with PD (20%) Abnormal BAEP wave morphology in patients with PD (53%)
Hawkins et al. (8)	Patients with PD (*n* = 40) HCs (*n* = 40)	oVEMPs, cVEMPs, SVV	Bilateral absence of BCV cVEMPs in patients with PD and HCs (15% vs. 0%, respectively) Significant reduction in BVC oVEMP amplitude in patients with PD than in HCs (13.20 μV vs. 16.04 μV, respectively) More abnormal SVV responses in patients with PD than in HCs (23/40 vs. 11/40, respectively)
Reichert et al. (11)	Patients with PD (*n* = 36) HCs (*n* = 316)	Bithermal caloric testing and ENG	Reduction or absence of caloric nystagmus in patients with PD (64%)
Venhovens et al. (12)	Patients with PD (*n* = 30) Patients with AP (*n* = 14) HCs (*n* = 25)	VEMPs, BAEPs, SVV, VNG	Patients with a history of falls (37%)
Vitale et al. (13)	Patients with PD with LTF (*n* = 11) Patients with PD without LTF (*n* = 11)	VNG	Abnormal vestibular examination results in all patients with PD with LTF (100%) Unilateral vestibular hypofunction in patients with PD without LTF (36%)
Di Lazzaro et al. (14)	Patients with PD (*n* = 15) Patients with PDPS (*n* = 15) HCs (*n* = 30)	cVEMPs	Unilateral absence of cVEMPs in patients with PD and PDPS (26% vs. 12%, respectively) Bilateral absence of cVEMPs in patients with PD and PDPS (6% vs. 40%, respectively)
Scocco et al. (15)	Patients with PD (*n* = 9) Patients with PDPS (*n* = 8) HCs (*n* = 18)	SVV	SVV deviations in patients with PDPS or PD
Gandor et al. (16)	Patients with PD without LTF (*n* = 9) Patients with PD with LTF (*n* = 21)	SVV	Normal SVV in patients with PD without LTF (100%) Pathological SVV in patients with PD with LTF (67%)
Bohnen et al. (17)	Patients with PD without FoG (*n* = 13) Patients with PD with FoG (*n* = 79)	Romberg test	Failure during condition 4 of the Romberg test in patients with PD without FoG (27.8%) failure during condition 4 of the Romberg test in patients with PD with FoG (84.6%)

AP, atypical parkinsonism; ACS, air-conducted stimuli; BCV, bone-conducted vibration stimuli; BAEPs, brainstem auditory-evoked potentials; cVEMPs, cervical vestibular-evoked myogenic potentials; FoG, freezing of gait; HCs, healthy controls; LTF, lateral trunk flexion; oVEMPs, ocular vestibular-evoked myogenic potentials; PD, Parkinson disease; PDPS, Parkinson disease with Pisa syndrome; SVV, subjective visual vertical; VNG: videonystagmography. ENG, electronystagmography; SOT, sensory organization test.

#### 2.1.1 Postural instability

Postural instability (PI) is one of the most distressing motor symptoms in PD and significantly increases the risk of falls (18, 19). Approximately 70% of patients with PD fall at least once annually (2, 20); moreover, this instability is known to increase with PD progression. PI occurs in approximately one-third of patients 2 years after the diagnosis of PD, rising to 71% after 10 years, and reaching 92% after 15 years (21). Vestibular signaling plays a crucial role in processing ego-motion information and regulating posture and balance (10), with vestibular dysfunction being an independent risk factor for falls in patients with PD and animal models (2–4). Six sensory integration tests using the NeuroCom Dynamic Postural Balance Instrument revealed that disrupted balance in patients with PD resulted from the inability to effectively interpret vestibular information independent of visual and proprioceptive integration and the loss of nigrostriatal dopamine during disease progression (3).

Altered vestibular-evoked myogenic potentials (VEMPs) increase as PD progresses to advanced stages and correlate with PI (22, 23), suggesting that impaired vestibular activity is a critical underlying factor. A prospective one-year follow-up study found that neurovestibular dysfunction might predict falls in at-risk patients with PD and imbalanced posture (12), suggesting that a baseline assessment using VEMPs may help predict future PI occurrence. Overall, PI in PD is associated with vestibular dysfunction.

Regarding anatomical localization, vestibular dysfunction in PD is associated with changes in the substantia nigra and pedunculopontine nucleus (PPN)–thalamic cholinergic innervation. For example, altered levels of vesicular acetylcholine transporters in the medial geniculate body are associated with PI and gait abnormalities in patients with PD (24). Cholinergic nerve endings in the vestibular brainstem nuclei include the caudal medial vestibular nucleus (25). Therefore, vestibular sensory information processing could be modulated by cholinergic signaling, further implicating vestibular dysfunction in PI development.

#### 2.1.2 Pisa syndrome

Pisa syndrome (PS), a disabling complication of PD, is defined as a specific set of recoverable postural changes involving ≥10° of lateral trunk flexion (LTF) and vestibular defects (13, 26, 27). The estimated prevalence of PS in patients with PD (PDPS) is 7.4–10.3% (28); these patients are more likely to have disordered balance and experience falls than those without PS (27). Therefore, PS severely reduces the QoL of patients and can lead to fractures and death, especially during advanced stages. As the primary system involved in regulating postural balance, vestibular dysfunction might be associated with PS development in patients with PD (29, 30).

Unilateral damage to the vestibulospinal reflex arc in experimental animals causes an imbalance in the descending motor regulation system of the spinal cord, resulting in scoliosis (31). Bilateral vestibulospinal reflexes are defective in patients with PDPS, who tend to have more severe cervical VEMP (cVEMP) abnormalities than healthy controls, suggesting an association between the vestibulospinal pathway and PS pathophysiology in PD (14). Scocco et al. (15) found that 14 of 21 patients with PD and LTF had pathological subjective visual vertical (SVV) perception; Gandor et al. (16) reported similar results. Therefore, vestibular balance disorders might be involved in LTF pathophysiology. Vitale et al. examined 11 patients with PD and LTF and found vestibular hypofunction in all of them (13). These findings suggested a potential connection between the postural changes in PD and a combination of vestibular dysfunction and altered somatosensory integration, with vestibular dysfunction driving the mechanisms leading to scoliosis in PD.

#### 2.1.3 Freezing of gait

Freezing of gait (FoG) is a unique and disabling clinical phenomenon characterized by brief episodes of inability to step forward or by extremely short steps that typically occur on initiating gait or turning while walking. Symptoms of FoG in patients with PD are also associated with vestibular dysfunction. Approximately 84.6% of patients experiencing FoG failed the modified Romberg 4 subtest, which focuses more specifically on altered vestibular function (17). Thus, vestibular processing deficits might pathophysiologically correlate with FoG in patients with PD.

Postural sensory processing, particularly of vestibular information, is poorer in patients with FoG, possibly due to impaired central processing of vestibular signals (32). Primates have a high vestibular response in the PPN (33). In addition, PPN connectivity can be enhanced via galvanic vestibular stimulation (GVS) in patients with PD (34), while rotational stimulation of the vestibular system might alleviate FoG (35). A study of four patients with PD who had undergone bilateral PPN and subthalamic (STN) electrode implantation found that deep brain stimulation (DBS) of the PPN improved vestibular perceptual thresholds, further confirming the response of PPN neurons to vestibular stimuli (36). Thus, the PPN might be a vestibular signal processing center within the brainstem, and its stimulation might improve posture and gait in patients with PD by modulating vestibular signals, thereby reducing the risk of falling. However, definitive proof of a causal relationship between FoG and impaired vestibular processing is lacking and further investigation is required.

#### 2.1.4 Eye movement abnormalities

The vestibular system controls not only gait and posture but also eye movements, mainly through the vestibular-ocular reflex (VOR), which detects head movements and maintains image stability on the retinal fovea. Pathological changes involving the formation of Lewy bodies induced by the immune response to α-synuclein, the vestibular nucleus, oculomotor nucleus, and basal and upper ganglia affect saccadic and microsaccadic eye movements (37). Coincidentally, evidence of VOR abnormalities have been reported in many early studies of PD (4, 38).

A study of eye movements in 35 patients with idiopathic PD using videonystagmography (VNG) revealed visual and vestibular motoneuron abnormalities (39). Similarly, patients with PD exhibited poorer stereopsis and impaired oculomotor behaviors, as assessed using a three-dimensional active shutter system and Tobii Eye Tracker, respectively (40). Large meta-analyses have also confirmed the occurrence of increased response latencies in pro- and anti-saccade tasks in patients with PD (41, 42). A cross-sectional study of oculomotor performance found prolonged saccadic latencies, poorer saccadic accuracy, and lower gain in smooth pursuit eye movement (SPEM) in patients with *de novo* PD compared with those in healthy individuals (43). In addition, vestibular rehabilitation (VR) was shown to help improve the VOR gain (44). Therefore, eye movement abnormalities in patients with PD appear to be closely associated with vestibular system changes. However, extensive case–control studies are needed to confirm this.

### 2.2 Associations between vestibular dysfunction and common nonmotor symptoms of PD

The impact of non-motor symptoms on the QoL of patients with advanced PD can be more significant than that of motor symptoms due to a lower treatment response. Some non-motor symptoms associated with vestibular dysfunction are dizziness, sleep and mood disorders, and cognitive issues.

#### 2.2.1 Dizziness

The prevalence of dizziness complaints among patients with PD (48–68%) is twice as high as that among older individuals (20–30%) (1, 45). A case–control study reported that patients with *de novo* PD sometimes experience dizziness for many years before disease diagnosis (46). Dizziness in patients with PD is most often attributed to orthostatic hypotension (OH) (47, 48). Accordingly, various cross-sectional studies have shown that 30–50% of patients with PD have OH (49, 50). However, many patients with PD without OH also complain of dizziness. This type of dizziness is called non-specific dizziness and refers to a symptom of feeling dizzy or lightheaded without a specific identifiable cause. Of note, non-specific dizziness is the second most prevalent type of dizziness after OH. A retrospective study including 80 patients with early-stage PD (disease duration ≤5 years) found that 37 (46.3%) presented with dizziness, which was non-specific in 11 (29.7%) of the cases (1).

Although the mechanism leading to non-specific dizziness in patients with PD is unclear, it may be affected by vestibular dysfunction. A prospective study of 84 patients with PD with and without OH found greater impairment of vestibular function in patients with dizziness compared with those without dizziness (51). An electronystagmographic assessment of 30 patients with PD found vestibular disorders in 83% of them, with a 43.3% prevalence of dizziness despite the absence of complaints (52).

In summary, dizziness is prevalent among patients with PD; the symptoms of non-specific dizziness might be closely associated with vestibular system dysfunction. Thus, targeted vestibular therapy or improving dysfunctional vestibular-related neural circuits might help relieve non-specific dizziness in patients with PD.

#### 2.2.2 Sleep disorders

Vestibular dysfunction might also be linked with sleep disorders. A study of nine adults with abnormal sleep patterns and shorter sleep durations uncovered greater bilateral vestibular hypofunction in these patients than in healthy controls (53). A cross-sectional study of 20,950 patients also found that 30% of individuals with vestibular vertigo had abnormal sleep durations; those with vestibular symptoms were more likely to experience insomnia or lethargy (54). Changes in VEMPs were found to directly correlate with rapid eye movement (REM), sleep behavior disorder, and PI (22). cVEMP abnormalities directly correlated with sleep scores in patients assessed using the REM Sleep Behavior Disorder Screening Questionnaire (55). Additionally, a small randomized controlled trial of 20 adults found that repeated electrical vestibular stimulation administered for 30 min/day improved total Insomnia Severity Index scores (56). These results indicated that vestibular pathways could project into multiple sleep and circadian-regulating nuclei in the brainstem and hypothalamus.

Mechanistically, the vestibular system regulates circadian rhythms and influences sleep behaviors by converging inputs from the visual and somatosensory systems (57). The higher centers of the vestibular system include many subcortical and cortical structures (58), which correlate with the function of the nerve center that regulates sleep; the hypothalamic circadian rhythm in animals is modulated through a neuroanatomical pathway between the medial vestibular nuclei and suprachiasmatic nucleus (59). In addition to sending projections to the cerebellar and brainstem nuclei, glutamatergic neurons in the vestibular nucleus project to the sleep–wake centers associated with brain regions that might be involved in sleep regulation (60).

Although the precise molecular mechanisms remain unknown, orexin expression increases after sleep deprivation (61). Interestingly, sleep regulation has been reported to depend on the activity of orexin-producing neurons in experimental animals. Orexin-producing neurons are closely associated with the vestibular system (62), supporting the correlation between the vestibular system and sleep regulation.

#### 2.2.3 Mood disorders

Mood disorders are a group of psychological disorders predominantly characterized by abnormal emotional regulation. Anxiety, fear, depression, and other neuropsychiatric symptoms are common in the general population and among patients with PD, although the underlying neurobiological mechanisms are complex and unclear (63). Many patients with impaired vestibular function also have emotional disorders, including those related to anxiety and fear (64). These relationships are not unidirectional, as mood disorders such as anxiety can lead to vestibular dysfunction and vice versa.

Ventricles synthesize adrenocorticotropic hormones, participate in processes associated with stress and anxiety, act on the vestibulolateral nucleus, and help regulate postural balance. Thus, stress, anxiety, and balance control are closely related to vestibular nuclei activity (65). Clinical findings have shown that caloric vestibular stimulation modulates mood and affective control (66); for instance, unrealistic optimism was selectively reduced during cold caloric stimulation of the left ear (67). The underlying mechanism might involve the vestibular cortex, and laterality may also be significant.

The right hemisphere is superior in dealing with negative emotions (68, 69). Similarly, vestibular cortical regulation is also affected by hemispheric lateralization, with the right hemisphere being more active than the vestibular cortex projection area in the left hemisphere in right-handed individuals (70). Hence, the right hemisphere might specialize in processes associated with interpreting or regulating negative emotions. More specifically, the right prefrontal region plays a role in negative emotional regulation associated with vestibular stimulation (71, 72). These findings indicated that cerebral hemisphere lateralization plays essential roles in vestibular and emotional processing, thus providing a theoretical basis for the interaction between the vestibular system and emotional processing centers.

#### 2.2.4 Cognitive challenges

Vestibular cognitive (also known as advanced vestibular) function is associated with visuospatial interaction, attention, executive function, and memory; accumulating evidence have supported a link between vestibular disorders and cognitive impairment in humans (73–75). Thus, cognitive decline in patients with PD might be associated with vestibular dysfunction.

Patients with PD exhibit visuospatial processing capacity deficits associated with aberrant neural circuitry in the frontal basal ganglia (76). However, this alone does not fully explain the cause of visuospatial deficits in PD. Similar to the basal ganglia, the vestibular system is also involved in spatial processing; vestibular dysfunction has been identified as the cause of decreased visuospatial competence in patients with PD (77). Patients with PD are more likely to experience cognitive dysfunction if they have gait and balance disorders; moreover, both symptoms worsen with disease progression (78). Dizziness might also be associated with cognitive decline in PD. For example, dizziness was closely associated with low Montreal Cognitive Assessment scores in patients with early-stage PD (1). Conversely, patients with refractory dizziness experienced significant improvement in cognitive function and dizziness-related indicators following VR therapy (79).

Vestibular function and cognitive processing might be linked to hippocampal activity (80). The hippocampus is involved in emotional processing closely associated with cognitive processes such as spatial memory (81). The human posterior hippocampus is involved in information processing and spatial memory, whereas the ventral hippocampus is responsible for emotional regulation (82).

The mechanisms underlying cognitive dysfunction in PD appear to be inextricably linked to the function of the vestibular system. However, further investigation is required to delineate these relationships.

#### 2.2.5 Other non-motor symptoms

Vestibular signal transmission and reception frequently coincide with those of other types of sensory information; central vestibular processing is promiscuous, contributing to its ubiquitous nature. Hearing loss has recently been recognized as an additional non-motor symptom of PD (83, 84). The factors affecting hearing loss are multifaceted, and common diseases such as otitis media with effusion also play a role in vestibular dysfunction (85). Hearing and balance function have certain neural connections and pathophysiologic mechanisms with motor control. Thus, neurodegeneration in PD may affect these common neural pathways, leading to hearing and balance problems. Although other non-motor symptoms, such as perception deficits, urinary problems, and cardiovascular and sexual dysfunction, are seemingly unrelated to vestibular function, some studies have suggested associations between these symptoms and VEMPs (7, 22). Thus, symptoms associated with vestibular system dysfunction are prevalent in patients with PD. While the underlying mechanisms are not fully understood, current pathologic, physiologic, and anatomic evidence support the idea that patients with PD have vestibular dysfunction (Table 2).

**Table 2:** Possible mechanisms of vestibular dysfunction in Parkinson’s disease (PD).

*Pathological aspects*
Parkinsonian neuropathological changes that occur in the vestibular nuclear complex, including the formation of Lewy bodies and Lewy neurites (5), reduced abundance of non-phosphorylated neurofilaments, and increased lipofuscin accumulation (6)
*Physiological aspects*
PD might include the modulation of vestibular nucleus excitability by dopaminergic systems (86). Small-conductance calcium-activated potassium (SK) channels expressed by dopaminergic neurons project directly to the vestibular nucleus (VN) and are involved in the homeostatic regulation of the vestibular system. Dopaminergic neurons in the substantia nigra are regulated by SK channels in rodent models of PD (87)
*Anatomical aspects*
PD includes reduced neuronal activity in the visual region of the cingulate gyrus on functional neuroimaging evaluations, which correlate with disease severity (88). The visual region of the cingulate gyrus is a key site for the integration of vestibular and visual inputs. (89). A study of balance and postural abnormalities in 10 patients with PD found that balance and postural control significantly improved after 8 weeks of upper extremity motor training, and functional imaging assessment revealed increased connectivity between the bilateral pallidum and the posterior part of the right cingulate gyrus (90). This suggests that vestibular information is conveyed to the cerebral cortex and might play a role in PD development. Some electrophysiological and neurotracer studies have also confirmed vestibular projections to the parafascicular thalamus (91, 92), suggesting that vestibular information is transmitted to the cerebral cortex and the striatum

## 3 Treatment options for relieving non-specific dizziness associated with vestibular dysfunction in patients with PD

### 3.1 Vestibular rehabilitation therapy

Although medications are indispensable for treating certain diseases, they can also cause serious side effects that might limit their use. In contrast, non-invasive VR therapy has been associated with better compliance than pharmacotherapy. Due to its consistently recognized effectiveness (93–95), it is now recommended in United States clinical practice guidelines (96). VR therapy can improve many PD symptoms, increasing gait speed, reducing dizziness, resolving balance disorders, and decreasing the frequency of falls (97–99). VR can also improve fatigue and enhance activities of daily living in patients with PD (100). VR works on the vestibular system through repetition of specific physical exercises that activate central neuroplastic mechanisms to achieve adaptive compensation of the impaired functions (101, 102). Synaptic inhibition or membrane hyperpolarization of neurons in the medial vestibular nucleus leads to a sustained increase in intrinsic excitability, a phenomenon referred to as “firing rate potentiation.” This mechanism could potentially be employed *in vivo* to facilitate behavioral plasticity (103, 104). Besides, balance training increases cortical thickness in visual and vestibular cortical regions, which favors vestibular compensation (105). In summary, a growing body of evidence has confirmed the positive effects of VR in patients with PD experiencing dizziness and balance disturbances, including (1) augmenting vision and proprioception to compensate for the vestibular loss, (2) developing compensatory strategies in situations of imbalance, and (3) developing substitution strategies to assist with gaze stability. Moreover, virtual reality and other technologies will continue to evolve and become more frequently applied in VR therapy, offering promising clinical applications.

### 3.2 Galvanic vestibular stimulation

The application of GVS for treating PD has attracted considerable attention because of its ease of management, non-intrusive nature, affordability, favorable safety profile, and minimal side effects. It is currently recognized as enhancing PD symptom improvement through vestibular-targeted therapy. Vestibular stimulation has been shown to result in the activation of multiple cortical regions (106), with GVS repeatedly achieving PD symptom amelioration (4, 107). Near-threshold stochastic vestibular stimulation can improve postural control in patients with PD (108, 109). Case reports have also suggested that suprathreshold GVS could improve postural reflexes (110), with repetitive caloric vestibular stimulation resulting in lasting motor and non-motor symptom improvement (111). Both noisy and sinusoidal GVS patterns can aid balance control by increasing the PPN connection amplitude (34).

Galvanic vestibular stimulation has also been used to treat dizziness (112) in patients with bilateral vestibular disorders. Significant neural activity in cortical areas involved in vestibular processing is associated with the severity of dizziness-related disability (113). The underlying mechanism likely involves a central role of the vestibular cortical network. These results were further validated in a functional imaging study (114). Therefore, GVS can not only help improve postural imbalance in PD, but also help reduce non-specific dizziness.

### 3.3 Repetitive transcranial magnetic stimulation

Repetitive transcranial magnetic stimulation (rTMS) is a non-invasive, painless, and non-destructive extracranial neuromodulation technology that has become the primary adjuvant therapy for PD in clinical practice (115), especially for complications arising from pharmacotherapies. Of note, rTMS has been shown to reduce motor symptoms and improve psychiatric symptoms, including anxiety and depression (116–118). Coincidentally, anxiety, depression, and stress commonly coexist with PD and can exacerbate dizziness symptoms. Psychological factors can amplify perceived dizziness severity and contribute to functional impairment. Therefore, rTMS may be effective in patients with PD who have non-specific dizziness, especially those with comorbid depression.

Moreover, rTMS has also been clinically applied to treat dizziness from other causes. A case study found that symptom severity was reduced by at least 50% after rTMS treatment over 3 months in a patient with chronic dizziness and persistent postconcussive symptoms (119). Eight women with a history of classic mal de débarquement syndrome were administered rTMS for 3 weeks in a prospective, double-blind, placebo-controlled study. They experienced significant improvements in various symptoms, including dizziness, mood disturbances, and anxious behaviors, achieving an approximate ten-point reduction in their Dizziness Handicap Inventory (DHI) scores (120). Therefore, we suggest that for patients with PD experiencing significant dizziness and depressive symptoms may benefit from daily sessions of rTMS lasting half an hour each, administered over a course of 1 to 3 months. This treatment approach could potentially help improve the patients’ symptoms of dizziness. However, the use of rTMS for treating non-specific dizziness in patients with PD has not been extensively studied. It is hoped that future large-scale clinical trials will be designed to confirm its effectiveness in this regard.

### 3.4 Deep brain stimulation

Deep brain stimulation (DBS) is an effective treatment for advanced PD (121–123). The relationship between DBS and vestibular function has recently attracted the interest of researchers. The cerebellum and vestibule work in tandem to assist in gaze stabilization and orientation for motion perception, thereby maintaining balance, with the cerebellum projecting directly to the ventroposterior and ventrolateral thalamus through the vestibulothalamic pathway (124). A study investigating DBS-induced changes in the thalamic ventral intermediate nucleus confirmed that SVV perception was altered during active electrical stimulation (125). DBS significantly improved vestibular discrimination accuracy and threshold in the rightward direction in patients with PD compared with that in healthy controls (126). Therefore, subthalamic DBS might exert differential effects on the vestibular and visual perception of linear motion in patients with PD. The effects of STN stimulation have also been investigated in a study of medial and caudal DBS electrode contact in five patients with PD (127). The patients described compromised rotational motion perception in the plane of the horizontal semicircular canal; one stated having the feeling of sitting on a swing. The latter form of complex perception impairment might be due to the combined stimulation of fibers from the vertical semicircular canals and otolith-derived signals, representing pitch and fore-aft motion, respectively. These findings provided new insights into the counterintuitive implementation of DBS for treating vertigo and imbalance caused by abnormal motion perception. Collectively, these studies suggested that treatment for enhancing vestibular function might be helpful for such patients.

## 4 Conclusion

Vestibular dysfunction is prevalent among patients with PD; however, the underlying mechanisms are poorly understood. Targeted treatment of vestibular-related symptoms might alleviate PD symptoms and enhance the QoL of patients.

Two major themes have emerged in the literature. One emphasizes the clinical challenges of vestibular dysfunction in PD, with an urgent need for better characterization of its symptoms. Vestibular dysfunction in PD may be linked to motor symptoms like PI, PS, FoG, abnormal eye movements, as well as non-motor symptoms such as dizziness, sleep disturbances, mood changes, and cognitive impairment. The second emphasizes what vestibular dysfunction reveals about PD regarding disease staging and distribution, and its role as an adjunctive assessment of PD prognosis. Further investigation in this area will have implications beyond the symptoms themselves to broader issues, such as non-specific dizziness in PD, cognitive decline, and developing new treatments for such symptoms in PD and other clinical situations. Future studies could use this as a starting point and focus on assessing whether targeted treatment of the vestibular system can delay PD progression, potentially facilitating the exploration of improved clinical approaches.

## Author contributions

MG: Funding acquisition, Methodology, Resources, Writing – original draft. LL: Data curation, Investigation, Methodology, Writing – original draft. LQ: Conceptualization, Data curation, Formal analysis, Writing – review & editing, Supervision. CW: Conceptualization, Investigation, Project administration, Validation, Visualization, Writing – review & editing.
